# Drowsiness Detection System Based on PERCLOS and Facial Physiological Signal †

**DOI:** 10.3390/s22145380

**Published:** 2022-07-19

**Authors:** Robert Chen-Hao Chang, Chia-Yu Wang, Wei-Ting Chen, Cheng-Di Chiu

**Affiliations:** 1 Department of Electrical Engineering, National Chung Hsing University, Taichung 40227, Taiwan; d106064401@mail.nchu.edu.tw (C.-Y.W.); darius.chen@siliconmotion.com (W.-T.C.); 2 Department of Electrical Engineering, National Chi Nan University, Nantou 54561, Taiwan; 3 Neurosurgical Department and Spine Center, China Medical University Hospital, Taichung 404332, Taiwan

**Keywords:** PERCLOS, drowsiness detection, sympathetic nervous index, parasympathetic nervous index, EEG

## Abstract

Accidents caused by fatigue occur frequently, and numerous scholars have devoted tremendous efforts to investigate methods to reduce accidents caused by fatigued driving. Accordingly, the assessment of the spirit status of the driver through the eyes blinking frequency and the measurement of physiological signals have emerged as effective methods. In this study, a drowsiness detection system is proposed to combine the detection of LF/HF ratio from heart rate variability (HRV) of photoplethysmographic imaging (PPGI) and percentage of eyelid closure over the pupil over time (PERCLOS), and to utilize the advantages of both methods to improve the accuracy and robustness of drowsiness detection. The proposed algorithm performs three functions, including LF/HF ratio from HRV status judgment, eye state detection, and drowsiness judgment. In addition, this study utilized a near-infrared webcam to obtain a facial image to achieve non-contact measurement, alleviate the inconvenience of using a contact wearable device, and for use in a dark environment. Furthermore, we selected the appropriate RGB channel under different light sources to obtain LF/HF ratio from HRV of PPGI. The main drowsiness judgment basis of the proposed drowsiness detection system is the use of algorithm to obtain sympathetic/parasympathetic nervous balance index and percentage of eyelid closure. In the experiment, there are 10 awake samples and 30 sleepy samples. The sensitivity is 88.9%, the specificity is 93.5%, the positive predictive value is 80%, and the system accuracy is 92.5%. In addition, an electroencephalography signal was used as a contrast to validate the reliability of the proposed method.

## 1. Introduction

In 2014, the National Highway Traffic Safety Administration (NHTSA), United States Department of Transportation **[1]** recorded 846 fatalities caused by drowsy driving, and this number is almost equal to the past 10 years. According to Finnish statistics, 16% of the fatal accidents involving connected vehicles are caused by fatigued driving [2]. Accordingly, accidents caused by fatigued driving can be reduced by developing a method to issue appropriate warnings to fatigued drivers.

Several studies have been reported regarding electrophysiological signals that can be used for drowsiness detection. For example, the HSV and mIIR algorithm was developed for the realization of the robust heart rate estimation system from webcam based face color image [3]. Qian et al. proposed a model of Bayesian non-negative CP decomposition (BNCPD) to extract common multiway features from the group-level electroencephalogram (EEG) signals and the average classification accuracy is 83.12% [4]. Fujiwara et al. proposed a drowsiness detection algorithm based on eight HRV features from electrocardiogram (ECG) [5]. The drowsiness was detected in 12 out of 13 pre-N1 episodes prior to the sleep on sets. However, their method requires drivers to place electrodes on the skin to measure ECG before driving.

Physiological signals can be measured using two types of methods: Contact and non-contact methods. The contact method involves the use of patches or mobile devices to facilitate measurement, including ECG and photoplethysmography (PPG), which utilizes photosensitive elements. The non-contact method has the advantages of a lack of contact with the human body and not making subjects feel uncomfortable. An example of the non-contact method is the photoplethysmographic imaging (PPGI) method, which uses webcam to receive light changes [6].

The drowsiness detection systems typically utilize blinking frequency or nod to make judgments, and rely on information that the driver has already entered a drowsy state, which is too late for the drivers to have a chance to react. The LF/HF ratio from HRV can be used to provide prewarning to drivers from drowsiness [7], but its accuracy is limited. This study proposed a drowsiness detection model system for simultaneously detecting using only a webcam, to cover all drowsiness levels, from slight to severe [8]. This study proposed a drowsiness detection system for simultaneously detecting the LF/HF ratio from HRV and eyelid closure using only a webcam, and a non-invasive PPGI signal can be obtained from face images [7]. Subsequently, pulse rate variability analysis (PRVA) was employed to determine the PPGI sequence, convert it to the frequency domain for analysis, obtain the sympathetic/parasympathetic balance index (LF/HF ratio), and determine the mental state of the subject. Thereafter, combined with the current face image, the position of the eyes was detected, and the drowsiness of the subjects was judged by the opening and closing of the eyes [9], thus increasing the robustness of the overall drowsiness detection system.

The existing methods used only one of the eyelid closure, ECG, PPG or EEG signals to detect the drowsiness. To the best of our knowledge, this study is the first one to combine both eyelid closure and PPGI signals to their advantage, thereby improving the accuracy and robustness of the drowsiness detection system. However, PPGI is obtained by a non-contact measurement of the facial image, the accuracy is inferior to conventional PPG. Therefore, the accuracy improvement of the proposed system is still limited. The advantages of the proposed method also include convenience, as no special equipment/helmet/clothing needs to be worn.

## 2. Technical Background

### 2.1. Physiological Signal of PPGI

PPGI is a non-invasive and non-contact method for measuring physiological signals using general video components, such as webcam and the image sensing components of mobile phones [3]. This method is based on the principle of the absorption and attenuation of light by blood vessels when light passes through the human body, and a change in the blood in the blood vessels with the beating of the heart [10]. Subsequently, the blood flows through the blood vessels of the skin and the changes in the absorption of light brightness can be converted into heart rate (HR) and heart rate variability (HRV) by observing the change in light [3,6].

Generally, video components are more sensitive to spectral recognition than the human eyes, and can recognize a wide range of wavelengths, including infrared (IR), near-infrared (NIR), and ultraviolet (UV) [6].

To employ the PPGI method, a dedicated light source is required, which is typically red light or NIR light [11]. The green light band exhibits a good absorption rate for heme, thus some studies use ambient light, including the green light band, as the light source [12].

**Figure 1:**
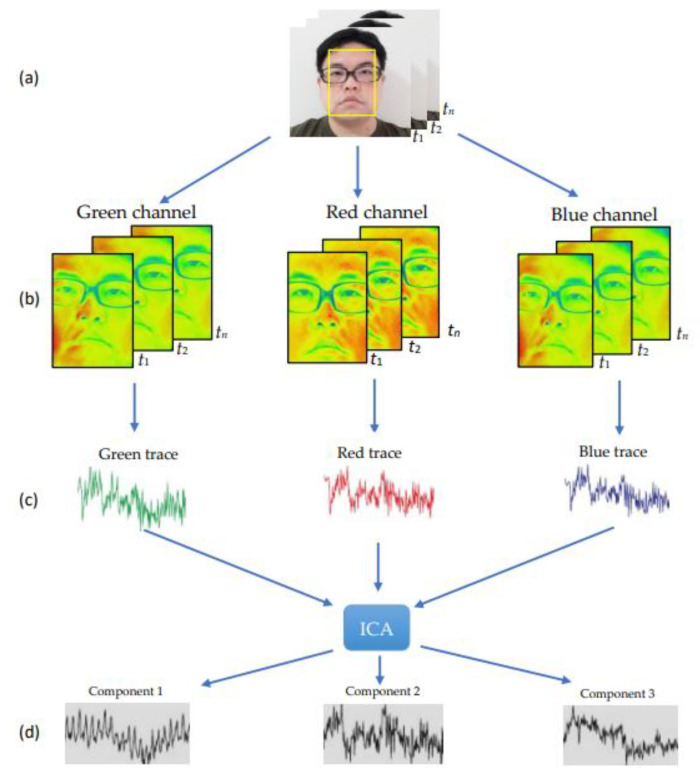
Schematic of photoplethysmographic imaging (PPGI). (**a**) Camera input image. (**b**) Perform RGB channel filtering on the input image. (**c**) PPGI signal obtained after RGB channel filtering. (**d**) Signals after ICA filtering.

The general PPGI process is shown in Figure 1. By selecting the region of interest (ROI) for the place of interest in each frame, the average change in the RGB pixels in the ROI can be calculated and the original signals of the three RGB channels can be obtained. In addition, more independent and clean signals for the three RGB channels can be obtained using independent component analysis (ICA) [13].

### 2.2. Heart Rate Variability Analysis

**Figure 2:**
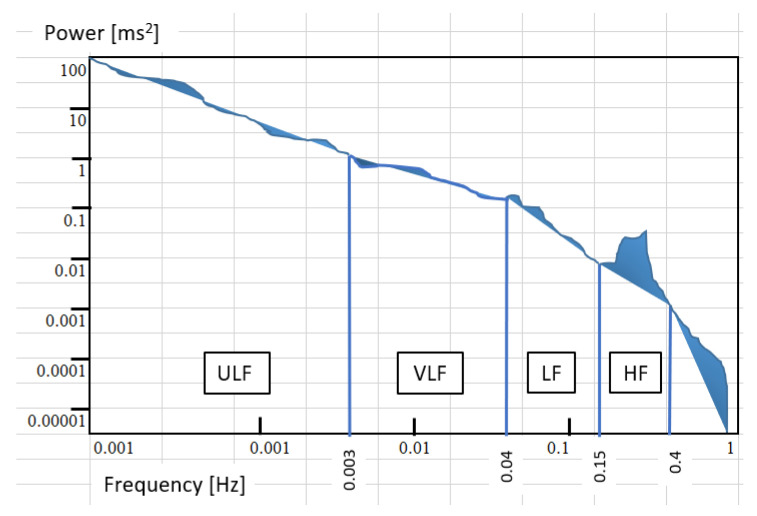
Frequency range definition on power spectral density (PSD).

The HRV is a measure of the degree of slight heart-to-beat variation during consecutive heartbeats. The heart rate changes due to complex physiological conditions and the regulation of autonomic nerves, and this subtle change is known as HRV [14]. Variations in the cardiac rhythm can be measured using two main methods: Time domain and frequency domain methods. For an ECG diagram, an R wave on two adjacent QRS complexes is defined as the R–R interval (RRI). As shown in Figure 2, the frequency domain analysis method involves the use of discrete Fourier transform or power spectral density (PSD) method to convert the time series of heartbeat intervals into the frequency domain. Early clinical trials revealed that the sympathetic and parasympathetic nerves in the autonomic nerve are related to the high and low frequencies in the frequency domain [15,16,17]. The clinical significance of each frequency domain is described in Table 1 [18].

### 2.3. PRVA

Heart rate variability analysis plays an important role in medicine. The basic waveforms and peak intervals of ECG and PPG were considered as RRI and PPI signals, and the rest and exercise states were marked as the control group. Moreover, the variability was analyzed. A previous study revealed that the PPG and ECG exhibited a good positive correlation in the resting state, indicating that PPG signal can replace ECG signal in the non-exercise state for the variability analysis [19,20]. Comparison of the mean and standard deviation of NN intervals (SDNN) between RRI and PPI can be seen in Figure 10 of ref. [20].

### 2.4. Autonomic Nervous System

The autonomic nervous system collectively refers to several physiological functions that cannot be consciously controlled [21], such as heart beat, respiration, blood pressure, digestion, and metabolism. The autonomic nervous system is mainly divided into two categories: Sympathetic and parasympathetic nervous systems. An enhanced sympathetic activity can result in an increase in the heart beat rate and blood pressure, a reduction in gastrointestinal peristalsis, an increase in pupil enlargement and metabolic rate, maintenance of alertness, and enhancement of concentration. The frequency domain analysis of sympathetic activity is mainly manifested at low frequencies. In contrast, an enhancement in parasympathetic activity can result in a decrease in the heartbeat rate, lower blood pressure, accelerated gastrointestinal peristalsis, constricted pupils, and sleep induction. In summary, the action of the parasympathetic nerve induces a relaxation and sleep state. Accordingly, in the frequency domain of HRV, the activity of the parasympathetic nerves is mainly expressed at high frequencies.

The autonomic nervous system requires the interaction of sympathetic and parasympathetic nerves to achieve regulation. An imbalance in this regulation will result in autonomic nervous system disorders, which induce strong effects, such as rapid increase in the blood pressure. In addition a strong parasympathetic nerve function results in a bad spirit feeling, laziness, tiredness, and physical decline.

Symptoms of autonomic dysfunction are systemic and do not necessarily act on the sympathetic or parasympathetic nerves. For example, although the quality of sleep is controlled by the brain, entering the sleep state depends on the regulation of the autonomic nervous system. In addition, as sympathetic nerves induce a nervous feeling, and parasympathetic nerves induce a relaxed feeling, one enters into a sleep state when the sympathetic nerves are weak and the parasympathetic nerves are strong. In contrast, when awake, the sympathetic nerves are stronger and the parasympathetic nerves are weaker.

### 2.5. LF/HF Ratio Analysis

Clinically, LF refers to sympathetic activity, and HF refers to parasympathetic activity. According to ref. [22], the LF/HF ratio differs among people, thus it is impossible to make a judgment based on a fixed threshold. The LF/HF ratio decreases with age. The LF/HF ratio of males in the younger age group (21–40 years old) is 2.2, almost double the amount of females (1.2). This may be attributed to the general belief that men are more active than women, and are more prone to anger, tantrums, and impulsiveness. The HRV is mostly measured during sleep when the HF is higher, thus the LF/HF ratio is typically less than 1.

### 2.6. Eyes Detection

Generally, before performing eye detection, a face search step should be performed. To this end, first, the position of the face should be determined, after which further eye detection is performed to determine the position of the eyes of the face. The template matching method is an example of face detection methods [23]. This method involved the establishment of a standard face template, after which the correlation coefficient between the template and the image is obtained. Typically, this method works better when the input image and training data have the same size, direction, and lighting conditions.

#### 2.6.1. Open and Closed Eyes Detection Method

This method involves the processing of the image that defines the position of the eye, and the determination of the open and closed states of the eye. Previous studies have proposed several methods to achieve this, such as color analysis method and angle method [24]. The color analysis method uses a less sensitive color space (HSV) to reduce the effect of light, in the HSV space, and the iris and the skin color can be clearly distinguished [25]. In addition, binarization is performed in the S domain to obtain the iris part, and the judgment of the opening and closing eyes can be made.

The angle analysis method involves the detection of multiple feature points of the eye, such as the corners of the eyes, the midpoint of the eye, and the midline of the upper eyelid, and defines the angles Degree (A) and Degree (B) [24] using these features. In addition, it utilizes the different angles of the opening and closing of the eyes to judge open and closed eyes.

#### 2.6.2. Percentage of Eyelid Closure over the Pupil over Time (PERCLOS)

PERCLOS, which is a NHTSA-approved drowsiness detection method, as listed in Table 2, is defined as the time occupied by a certain percentage (70% or 80%) of the eyes closed in a unit time (usually 1 min or 30 s), and drowsiness is considered to have occurred when the above conditions are met. As listed in Table 3, there are currently three commonly used indicators of closed-eye pupil coverage [25]. According to the US Highway Traffic Safety Administration and other drowsiness detection related experiments, it is believed that P80 exhibits the highest correlation with the degree of drowsiness, thus most studies have focused on P80.

### 2.7. Brain Wave Analysis

Brain waves are physiological signals produced by the human body via the communication of the billions of neurons in the human brain by the transmission of electrical signals. The reflection of brain neurons during activity appears as a wave on an EEG. The analysis of the physiological and psychological states of humans through changes in brain wave signals has been widely used in various fields and is currently considered to be the most direct and accurate method to judge awareness.

The EEG is a continuous curve graph that measures the electrical activity of the cerebral cortex, and receives and amplifies it. In every moment, regardless of the activity, the vibration frequency of the brain waves of humans differs significantly when they open their eyes, close their eyes, wake up or fall asleep. The International Organization of Societies for electro-physiological technology classifies brain waves into Alpha (*α*), Beta (*β*), Delta (*δ*), and Theta (*θ*) waves according to their frequencies [26]. The frequency range of delta waves (*δ*) is <4 Hz. In adults, delta waves are typically observed during nonrapid eye movement (NREM) sleep [27]. The frequency range of theta (*θ*) wave is 4 to 7 Hz. Occasional theta or even delta frequencies (1–3 Hz) may be observed during normal wakefulness, mainly over the temporal regions [28]. This awake theta may be generated in adults during emotional processes, such as anger or fear [29,30,31], during a split-attention state due to multitasking or multisensory conditions [32], in a state of mental fatigue [33] or during the performance of memory tasks [34]. However, theta activity only becomes prominent during drowsiness. The frequency of the alpha (*α*) rhythm is 8 to 13 Hz and this activity is typically seen during normal EEG recordings in the occipital region of awake adults. The alpha rhythm is best seen with eyes closed and mentally relaxed, and is noticeably reduced with eyes open and mental effort [27]. The frequency of beta (*β*) rhythms is 14 to 30 Hz and their amplitude is usually small [27]. This amplitude often increases during drowsiness [27]. Beta is most prominent over the central and frontal EEG channels and is less seen over posterior regions of the scalp [27]. Most sedatives (e.g., benzodiazepines and barbiturates) increase the overall abundance and amplitude of beta activity [35].

Brain computer interface (BCI) utilizes physiological signals and brain waves sent by non-muscle channels. When the brain is active, it produces specific brain wave changes, thus the brain wave can be monitored using a brain wave instrument. Traditionally developed non-invasive equipment requires the subject to wear a special headgear, which comprises of numerous electrode pads in contact with the scalp for measurement; however, this device cannot be easily used for daily life measurement, and is more inclined to be used during in-depth examinations in hospitals. There are several wireless portable brain wave measurement devices that have been developed, such as the MindWave Mobile [36]. The brain wave signal analyzed using the contact-type brain wave detection device was used as an important experimental control group index.

## 3. Proposed System Architecture

### 3.1. System Architecture

**Figure 3:**
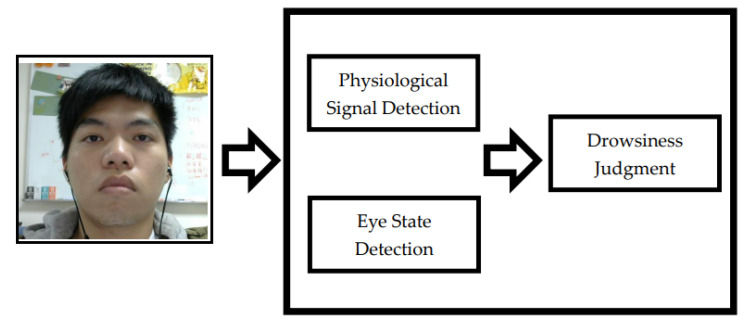
Algorithm block diagram of the drowsiness detection system.

The proposed system architecture combined physiological signals detection with PPGI signal to obtain LF/HF ratio from HRV and human eye tracking to increase the accuracy and robustness of drowsiness detection. Particularly, it only uses the face images obtained by webcam to achieve a non-contact drowsiness detection system. As shown in Figure 3, the structure of the algorithm consists of three parts: Physiological signal detection, human eye state detection, and drowsiness condition judgment.

**Figure 4:**
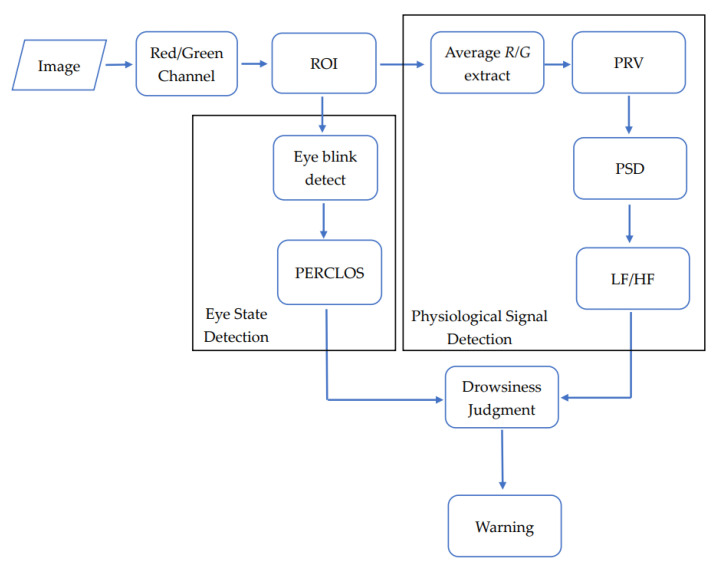
Drowsiness detection system diagram.

As shown in Figure 4, after acquiring the face image, first, the brightness of the image environment is detected to determine the color channel, after which the ROI for the place of interest in the image is determined. In terms of physiological signals, the average value of the channel in each frame ROI was calculated, and the original PPGI signal can be obtained, and P wave detection can be performed to obtain the PPI sequence. The LF/HF ratio was obtained by performing spectrum conversion on the PPI sequence. To derive the state of the human eye, the image of the eye block was obtained from the ROI, after which the image was preprocessed to obtain the position of the iris for the eye opening and closing detection, and the obtained eye opening and closing state was used to calculate the PERCLOS. Finally, the two results were combined for drowsiness judgment and statistics.

### 3.2. Red/Green Channel of PPGI

Generally, the best absorption rate of heme is observed in the green light band, thus a better original signal waveform can be obtained using the Green channel; however, the Green channel is easily affected under poor light conditions. Therefore, it is necessary to employ an NIR light source to enhance illumination to increase the image recognition rate. When the infrared light source is used to enhance illumination, the Red channel can be used to obtain the change amount of the infrared absorption of the heme, which will exhibit a better original signal waveform. The input format of webcam used in this study was *YCbCr* format, which is a representation of a color space, where *Y* stands for Luminance, and *Cb* and *Cr* stand for Chrominance. This format is convenient for separately calculating chrominance. Equation (1) shows the *YCbCr* and *RGB* conversion formula.

(1) $$\left[\begin{array}{c} Y\\ Cb\\ Cr \end{array}\right]=\left[\begin{array}{ccc} 0.299 & 0.584 & 0.114\\ -0.169 & -0.331 & 0.5\\ 0.5 & -0.419 & -0.081 \end{array}\right]\left[\begin{array}{c} R\\ G\\ B \end{array}\right]+\left[\begin{array}{c} 0\\ 128\\ 128 \end{array}\right]$$

**Figure 5:**
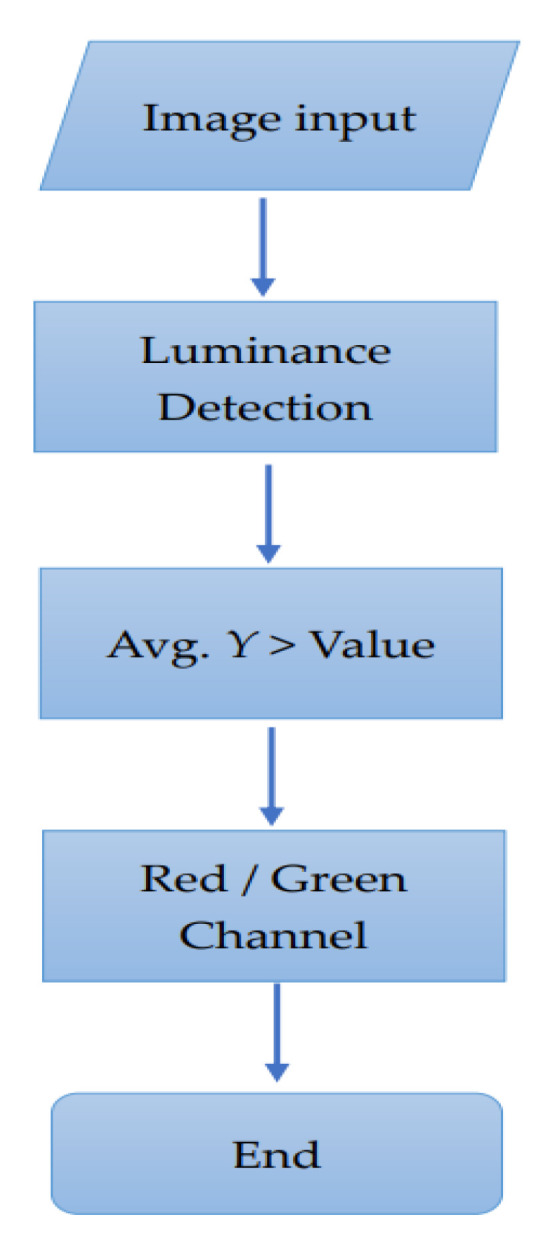
Red/Green channel selection flow chart.

As shown in Figure 5 using the characteristics of the *Y* (brightness) image, first, we calculated the average brightness of the input image. If the average brightness was higher than the set standard value, the Green channel was used, whereas if it was below the standard value, the Red channel was used.

### 3.3. Determination of the ROI

The detection of physiological signals and the human eye states requires the subsequent processing of different blocks of the face image. The eye detection algorithm used in this study was based on a previous study [37], which described face detection and eye search methods.

**Figure 6:**
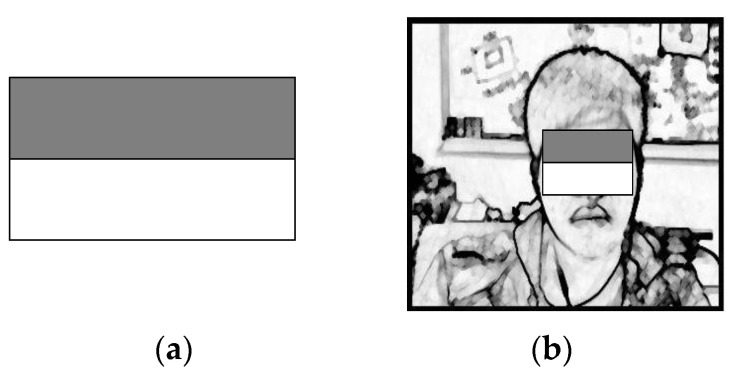
Haar-like feature 1. (**a**) Model-1. (**b**) Search chart.

As shown in Figure 6a, after the pre-processing of the input image is completed, a feature search of scale 1 is performed, and one of the features of the Haar-like feature is used here. As shown in Figure 6b, this feature was used as a template comparison program after completion.

**Figure 7:**
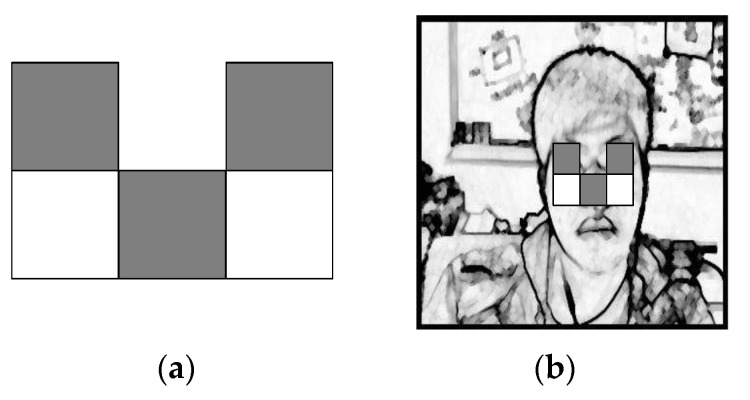
Haar-like feature 2. (**a**) Model-1. (**b**) Search chart.

As shown in the template in Figure 7a, after obtaining the scope of scale 1, a feature search was performed on scale 2 using the template to obtain the face scope, including eye information, and the search result is shown in Figure 7b.

**Figure 8:**
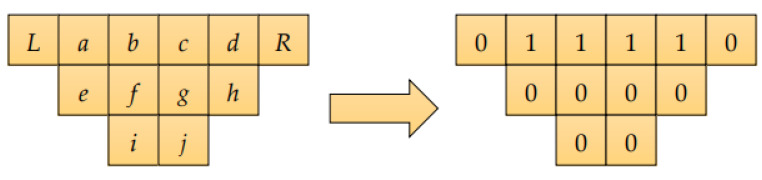
Inverted triangle template.

**Figure 9:**
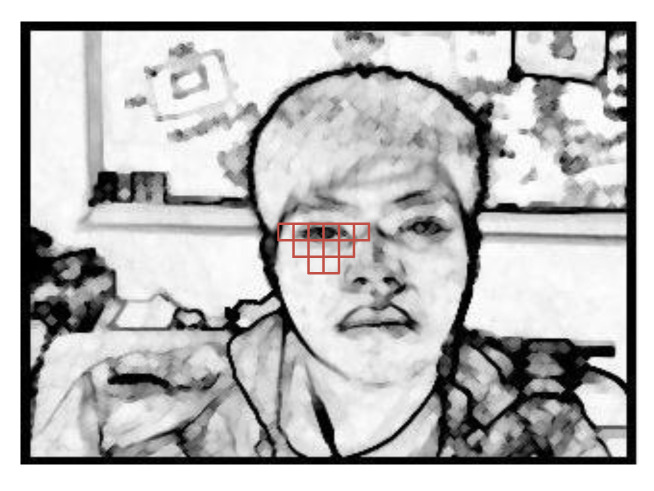
Inverted triangle template search diagram.

Using the LGP method [38], the design was divided into 12 equal parts of an inverted triangle template for eye range search. In Figure 8, the average value of (*e*, *f*, *g*, and *h*) was calculated and used for each block. The difference is 1 if the difference is less than the average value, and 0 if the difference is greater than the average value. The samples can be considered the same regardless of whether the eyes are open or closed. As shown in Figure 9, the eye block range can be determined.

**Figure 10:**
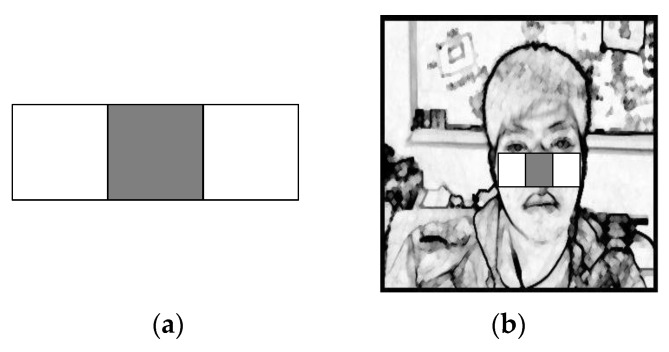
Haar-like feature 3. (**a**) Cheek search template. (**b**) Search chart.

For the ROI required by the physiological signal, we selected the cheek, which exhibits a larger area and thinner skin, and a better effect for obtaining the PPGI signal. As shown in Figure 10a, in the scale-2 feature search, the template-2 model is used instead. The search result is shown in Figure 10b, and the desired block was obtained.

### 3.4. PRVA

The PRVA mainly consists of two parts: The acquisition of the PPI sequence and the conversion of the power density spectrum, which functions to mainly convert the PPGI signal into a spectrum to obtain the LF/HF ratio.

#### 3.4.1. PPI Detection

**Figure 11:**
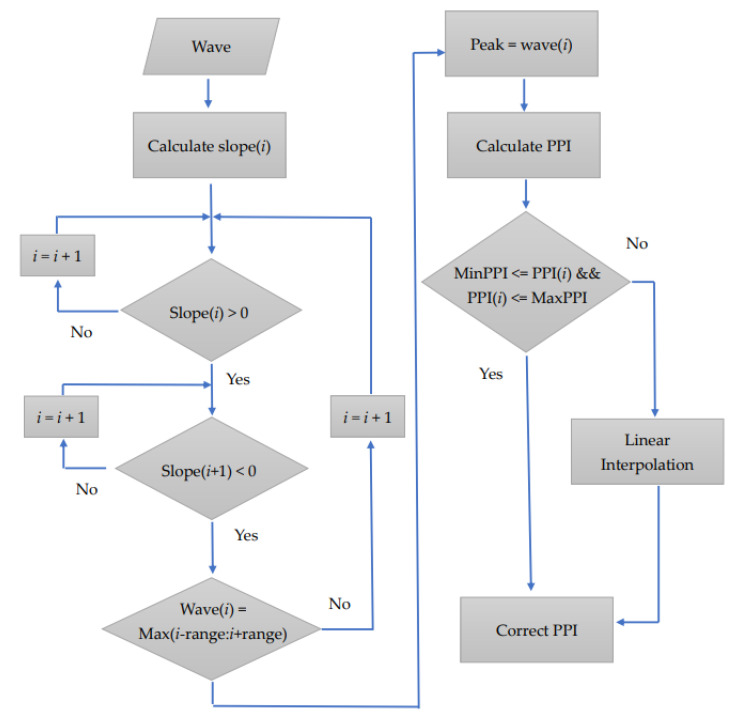
PPI detection flow chart.

**Figure 12:**
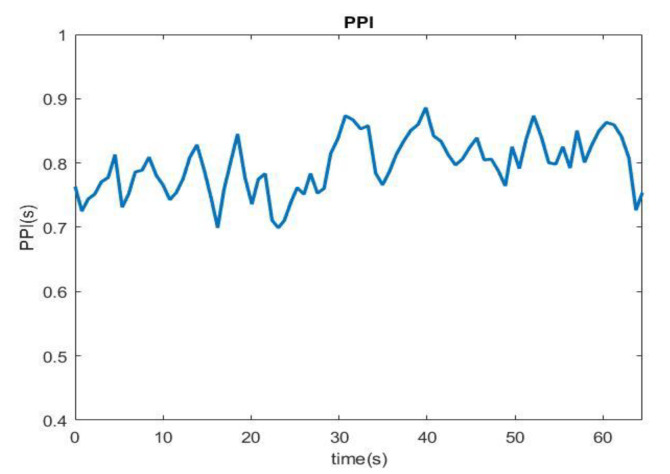
PPI sequence diagram.

The PPI detection method used in this study is a modification of the application technology principle employed in a previous study [9]. The PPGI signal obtained through the webcam first passes through the bandpass filter to preferentially filter out the extra noise generated by the input of the hardware system. PPGI usually has limited temporal precision, the minimum exposure time required and the large amount of data led us to set the frame rate below 25 fps. The PPI signal was then extracted with signal preprocessing, including basic digital signal theory, Ectopic replacement to resample, zero-mean and window function process. Then, we set a search peak interval range and the normal range of the human heartbeat to define the upper and lower limits of PPI to exclude abnormal signals caused by noise. The peak was determined using the slope method, and the slope of the current position was calculated. The peak point can be observed with a change in the slope value from a positive value to negative value. To confirm whether the value is the maximum value, the previous range was searched. If it is the maximum value, it is considered as the peak value. The PPI sequence can be obtained by subtracting the time points of each peak. Figure 11 shows the flow of the PPI detection method. Next, whether the PPI sequence exceeded the defined upper and lower limits of the PPI was examined. When the PPI exceeded the normal range, the closest normal PPI before and after was obtained to perform linear interpolation, after which the abnormal PPI was replaced. To obtain the PPI sequence, first, each peak of the PPGI signal was determined. After the signal was analyzed by PRV, the PPI sequence was obtained, as shown in Figure 12.

(2) Slope(i)=−2X(i−2)−X(i−1)+X(i+1)+2X(i+2)

#### 3.4.2. Power Density Spectrum

In the variability analysis, the mental state was reflected in the frequency domain analysis, but when the signal was undergoing spectrum conversion, a Gibbs phenomenon, which will result in severe distortion of the high and low frequency signals, occurred. The PRV analysis is mainly the change of the low frequency signal, thus the error suppression of the FFT must be minimized. As a result, the error was reduced using the Hanning Window in the window function.

Frequency domain analysis utilizes a short RRI to perform spectrum analysis, and quantifies the corresponding range of the autonomic nerve using different frequency bands. This study selected a relatively simple algorithm and fast processing speed (fast Fourier transform).

### 3.5. Open and Closed Eyes Detection

Using the ROI setting method, we obtained the block image of the eye area, after which the block image was processed to obtain the open and closed states of the eyes.

After inputting the eye block image, we utilized the edge image to identify the eye to measure its size and cut the image. Edge detection is a type of high-pass filter of the gradient calculation method. After binarization, the edge part of the eye was highlighted, and the entire edge map was calculated as a value square, and the average value of the edge is derived as the threshold, after which Hough transformation was used to determine the most circle-like place to determine the iris position and iris radius (*Rs*). When the position and radius of the iris are determined, the eye can be open and closed, and the brightness value of *Rs*/2 size outside the iris edge was subtracted from the brightness value of *Rs*/2 size inside the iris edge. As illustrated in Equation (3), when the eyes are open, the difference between the outer circle and the inner circle is greater than when the eyes are closed, and the eye open value is obtained.

(3) $$\mathrm{Openvalue}=\mathrm{abs}\left(\sum_{Rs}^{\frac{3}{2}Rs}intensity-\sum_{\frac{1}{2}Rs}^{Rs}intensity\right)$$

#### 3.5.1. Enter Sleep State

The falling asleep point was determined using the rapid eye movement (REM) in the electromyogram and the *α* wave in the brain wave. When a person enters the sleep state, a REM cycle begins. With an increase in the REM cycle to 2, the *α* wave appears, and can be judged as the time point when you really fall asleep [39]. The LF value decreases significantly during this period, and the LF/HF ratio also decreases. This time is defined as the Sleep Onset.

#### 3.5.2. Judgment Conditions

(1) Condition 1: LF/HF ratio_(Now)_

LF/HF ratio_(Now)_ refers to the current sympathetic/parasympathetic balance index. First, we calculated the mean LF/HF ratio for the first 5 min (LF/HF ratio is related to gender and posture). As shown in Table 4, condition 1 is divided into six levels using Equations (4) and (5). An increase in the grades from small to large (1–6) corresponds to a higher sleepiness tendency, and condition 1 refers to the comparison of the current LF/HF ratio of the six grades.

(4) $$\mathrm{Mean}\left(Y\right)=\frac{1}{N}\overset{N}{\underset{i=1}{\sum }}Y_{i}$$

where *Y* is the LF/HF ratio, and *Y_i_* is the *i* group of data with a total of *N* groups.

(5) $$X_{i}=\left(\mathrm{Min}+0.1\right)+\frac{\mathrm{Mean}\left(Y\right)-\left(\mathrm{Min}+0.1\right)}{5}i\;\;\;\;\left(\;i=0\;\sim \;5\;\right)$$

(2) Condition 2: LF/HF ratio_(Now)_—LF/HF ratio_(Avg)_

Condition 2 was considered to calculate the average LF/HF ratio (LF/HF ratio_(Avg)_) for 2 min, and compare it to the LF/HF ratio_(Now)_. As shown in Table 5, this condition was divided into five grades using standard deviation (SD). An increase in the scale value from small to large (1–5) corresponds to a higher tendency to doze off.

(6) $$\;\mathrm{SD}\left(\mu \right)={\sqrt{\frac{1}{N}\overset{N}{\underset{i=1}{\sum }}{\left(X_{i}-\mu \right)}^{2\;}}}_{}$$

(3) Condition 3: PERCLOS

The sampling frequency of webcam in this experiment was 30 frames/s, indicating that the number of frames detected in 1 min was 270. As shown in Table 6, we divide the condition of PERCLOS into three levels, and the drowsiness characteristics increased with an increase in the level.

#### 3.5.3. Drowsiness Judgment Analysis

To obtain the drowsiness index, condition 1 and condition 2 were comprehensively judged. As shown in Table 7, the drowsiness indicators “1” to “3” represent a higher tendency to feel drowsy.

The experiment generated an LF/HF ratio every 4s. Through the comprehensive judgment of condition 1 and condition 2, the sleep index can be obtained. In addition, 30 drowsiness indexes were obtained by performing statistics for 2 min and overlap for 1 min. The condition classification is shown in Table 8. According to the obtained 30 indicators, the drowsiness level was divided into three.

Previous experiments were performed using the drowsiness judgment condition of PPGI signal. The final comprehensive judgment was performed by comparing the drowsiness level and the condition 3 PERCLOS was used to obtain the warning value. As shown in Table 9, the warning value “0” represents Awake, the warning value “1” represents Fatigue, and the warning value “2” represents Drowsy. When both drowsiness level 1 and condition 3 level 1 appear twice in a row, the warning value “1” will be changed to “2”. This was performed to reduce misjudgments and improve early warning capabilities.

## 4. Experimental Evaluation

### 4.1. Experimental Setting

The proposed system utilized ZedBoard, which is based on the Xilinx Zynq™-7000 extended processing development platform, and is equipped with a webcam equipped with an NIR light source, and a brain-computer interface MindWave Mobile [36] was used as the sensor device. The software interface adopted Qt Creator, which provides a visual debugging tool and an integrated GUI layout, which is convenient for accelerating the development process, and integrating the input and output control functions of webcam.

### 4.2. Webcam Specifications and Controls

The specification of the NIR webcam used in this experiment is shown in Table 10. This webcam can actively emit NIR light sources when the light is insufficient. A standard UVC (USB video device class) device was used as the webcam, and it can be referred to V4L2 (Video4Linux2) driver firmware. V4L2 is the API for video capture and video output. Through V4L2, you can receive webcam images in real time. First, the received image data were stored in the DDR memory, after which the data were read in *YCbCr* format. Finally, the read data were processed using our algorithm, and the results were obtained after the image was output to the GUI interface.

### 4.3. Experimental Flow

**Figure 13:**
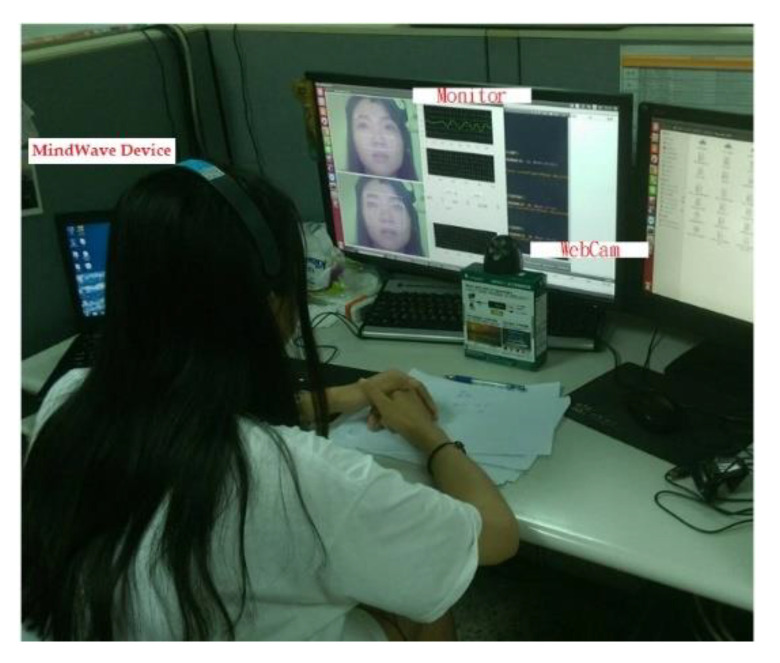
Setup of the experimental measurement.

According to the standard set by the European and American Heart Association [40], HRV analysis can be divided into long-term and short-term. The short-term recording is at least 5 min, and can also be 10 and 20 min. The long-term is mainly 24 h. This analysis mastered and recorded the process of the mental state of the subject from normal to drowsy. The measurement diagram is shown in Figure 13. The subjects in this study were six men and two women, aged between 20 and 25 years old. The participants were healthy, voluntary, and cognitively tested during the assessment. They had given informed consent. They were approximately 40 to 50 cm away from the webcam and wore the brain wave monitor MindWave Mobile [36] to simultaneously measure the EEG signal, as a reference standard. The experiments were conducted around 1:00 pm. There were about 1200 min of recording in total. The experiments totally collected 40 samples, including 10 awake samples and 30 sleepy samples.

### 4.4. Experimental Results

This study utilized EEG signals as the benchmark of the simulation results. The brain wave was measured every second using a brain wave instrument, and at the end of the measurement, the brain wave information measured every second was recorded in *α*, *β*, *δ*, and *θ* waves, and provides information to people. The algorithm for measuring the current state, after calculating the collected brain waves, obtained the concentration (Attention) and the degree of relaxation (Meditation) with an index of 0 to 100.

**Figure 14:**
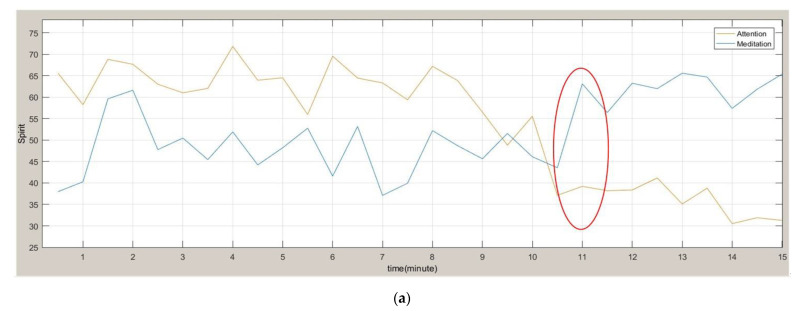

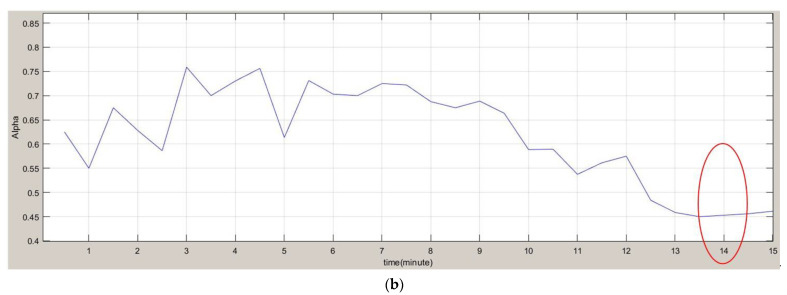
Comparison of attention, meditation, and alpha waves. (**a**) Brain waveforms of attention and meditation. (**b**) Alpha waveform.

The concentration and relaxation of 30 strokes were averaged and updated every 30 s. The simulation results are shown in Figure 14a. When the concentration was lower than 40 and the relaxation was greater than 60, this indicates that the mental state of the subject has declined and a warning is issued. The results revealed that before the Alpha wave value dropped to the point of falling asleep, the concentration and relaxation values reached the warning standard (Figure 14b).

**Figure 15:**
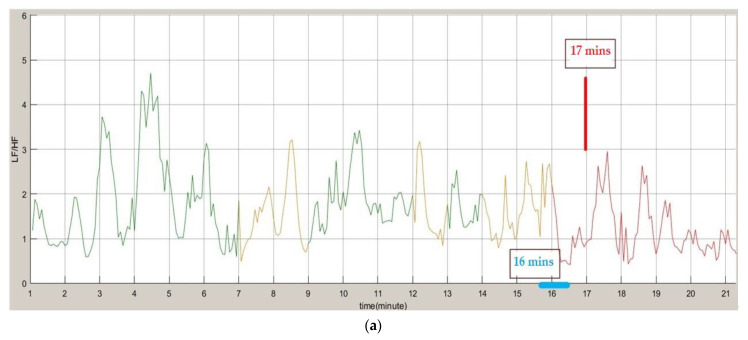

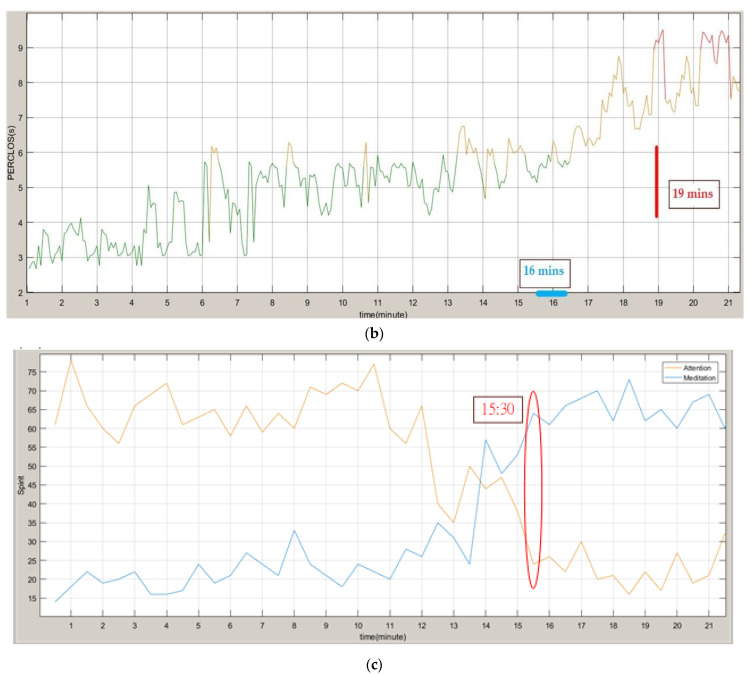
Experimental results of awake to sleepy. (**a**) LF/HF drowsiness judgment. (**b**) PERCLOS result. (**c**) Brain wave analysis results.

The LF/HF ratios and PERCLOS information and brain waves obtained using our drowsiness detection system were analyzed. Both the LF/HF and PERCLOS were updated once every minute, and the brain waves were updated every 30 s. Figure 15 shows the results of the subjects from being awake to dozing off (a) is the result of LF/HF analysis obtained through dozing judgment, (b) is the PERCLOS result, and (c) is the brain wave analysis result.

The results revealed that LF/HF issued a warning at the 17th min, and PERCLOS issued a warning at the 19th min. However, when we performed a comprehensive judgment of drowsiness, we observed that both LF/HF and PERCLOS appeared at the 16th min. This corresponded to a fatigue state, thus in our system, a warning can be issued at the 16th min to achieve a better early warning effect. In addition, it was observed that the brain wave started to change significantly from the 12th min, and a warning appeared at the 15th min, 30th s, which is consistent with our experimental results.

**Figure 16:**
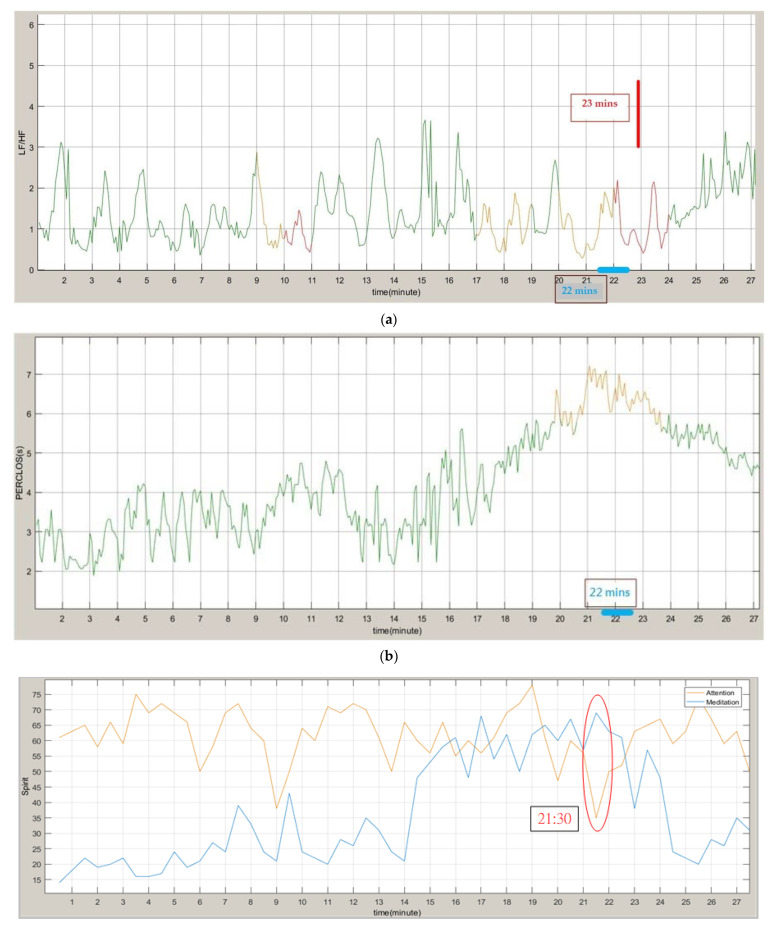
Experimental results after the addition of an alarm bell. (**a**) LF/HF drowsiness judgment. (**b**) PERCLOS result. (**c**) Brain wave analysis results.

We expected that an alarm bell will remind the subjects to cheer up when the subjects are detected to be in a dozing state. Figure 16 shows the experimental data after the addition of an alarm bell. In the LF/HF drowsiness judgment, a warning was issued at 11 and 23 min. Compared to the brain wave analysis results, the 11 min warning may be a misjudgment, but in the comprehensive judgment of drowsiness with the PERCLOS results, our system will not be able to judge at 11 min. A warning can be issued, and a warning can be issued at 22 min, which is closer to the warning time analyzed by the brain waves. After the warning sound was triggered, changes were observed. The LF/HF value gradually increased, the amount of PERCLOS closed eyes decreased, and the brain wave analysis decreased significantly, indicating that the use of the warning sound exhibited a significant effect on returning the subject to an awake state.

The accuracy of the system for the 40 samples was analyzed, and the result is presented in Table 11. The sensitivity is 88.9%, the specificity is 93.5%, the positive predictive value is 80%, and the system accuracy is 92.5%.

## 5. Conclusions

This study proposed a non-contact drowsiness warning system based on facial images combining the detection of LF/HF ratio from HRV of PPGI and PERCLOS. This warning system reminds the driver to reduce the risk of traffic accidents when the driver feels fatigued due to long-term driving. To enable the practical application of the system in various environments, this system utilized an NIR camera as an active light source device. In addition, the system requires information on the color when obtaining PPGI. Furthermore, the influence of light changes was reduced by selecting Red/Green channels. The eye status was acquired using gray-scale images without color information to ensure that the proposed drowsiness detection system can be used day and night.

The drowsiness detection system proposed in this study was divided into two subsystems, detection of LF/HF ratio from HRV of PPGI and detection of human eye state. In this study, PPGI was employed to obtain physiological signals in order that the system can issue warnings in advance; however, it is easily affected by external light, which results in misjudgment results. PERCLOS exhibits a high accuracy for the detection of eye opening and closing; however, it cannot issue a warning in advance. This study combined the advantages of the two subsystems and compensated for the shortcomings of both to achieve a more robust and accurate system.

In future work, combination of different physiological signals can be further investigated to assist in judging drowsiness and improving the system performance. Moreover, deep learning algorithms will be incorporated into the proposed system to increase accuracy. Additional test cases will be collected, based on classification conditions, such as gender, age, profession, differences in the external environment, etc., and compared with standard EEG to further thoroughly test and improve the detection system.

## Figures and Tables

**Table 1:** Frequency range definition.

Index	Definition	Clinical Significance
Total Power, TP (ms^2^)	Set the sampling frequency to ≤0.4 Hz, and sum the variation frequency bands of all normal heartbeat intervals	Global Heart Rate Variability Assessment
Very Low Frequency Power, VLFP (ms^2^)	Set the sampling frequency to ≤0.04 Hz, and the variation in the normal heartbeat interval in the very low frequency range	Physiological significance is unknown
Low Frequency Power, LFP (ms^2^)	Set the sampling frequency to 0.04~0.15 Hz, and the variation of the normal heartbeat interval in the low frequency range	Sympathetic nerve activity
High Frequency Power, HFP (ms^2^)	Set the sampling frequency to 0.15~0.4 Hz, and the variation of the normal heartbeat interval in the high frequency range	Parasympathetic nerve activity
LF/HF	The ratio of high and low frequency power	Balance of autonomic nervous activity

**Table 2:** PERCLOS drowsiness definition.

Classification	PERCLOS Value
Awake	PERCLOS < 0.075
Questionable	0.075 < PERCLOS < 0.15
Drowsy	PERCLOS > 0.15

**Table 3:** PERCLOS index definition.

PERCLOS Index	Definition
P70	Pupil coverage over 70%
P80	Pupil coverage over 80%
EM	Pupil coverage over 50%

**Table 4:** Condition 1 range.

Mode	Range
1	*X*_5_ < Ratio
2	*X*_4_ < Ratio < *X*_5_
3	*X*_3_ < Ratio < *X*_4_
4	*X*_2_ < Ratio < *X*_3_
5	*X*_1_ < Ratio < *X*_2_
6	*X*_0_ < Ratio < *X*_1_

**Table 5:** Condition 2 range.

Mode	Range
1	*β*_1_ × SD < Diff
2	*β*_2_ × SD < Diff ≤ *β*_1_ × SD
3	*β*_3_ × SD < Diff ≤ *β*_2_ × SD
4	*β*_4_ × SD < Diff ≤ *β*_3_ × SD
5	Diff ≤ *β*_4_ × SD

**Table 6:** Condition 3 range.

Mode	Range
0	PERCLOS < 180
1	180 < PERCLOS < 270
2	270 < PERCLOS

**Table 7:** Condition 1 and condition 2 judgment.

	Condition 2	1	2	3	4	5
Condition 1						
1		1	1	1	1	1
2		1	1	1	2	2
3		1	2	2	2	2
4		2	2	2	3	3
5		2	3	3	3	3
6		3	3	3	3	3

**Table 8:** Level of drowsiness.

Drowsiness Level	Condition
0	1. When the previous level 2 is level 0, the judgment is changed to level 0.2. When the proportion of indicator 1 appears ≥66%.
1	1. When index1 is not found.2. When the proportion of index 3 is ≥33% and the total proportion of index 2 is ≥50%.
2	1. When the proportion of index 3 is ≥66% and the total proportion of index 2 is ≥80%.2. Change the judgment to level 2 when three levels of level 1 are observed in a row.

**Table 9:** Drowsiness warning form.

	Condition 3	0	1	2
Level				
0		0	0	1
1		0	1	2
2		1	2	2

**Table 10:** Webcam specifications.

Chipset	SMI
Resolution	640 × 480
Output format	*YCbCr*422
Infrared wavelength	850 ± 50 nm
Output interface	USB 2.0
Sample rates (kHz)	30

**Table 11:** System efficiency indicator.

	Predicted	Awake Judgment	Doze Judgment
Actual			
Wide awake		8	1
Doze		2	29
Sensitivity is 88.9%			
Specificity is 93.5%			
Positive predictive value is 80%			
System Accuracy is 92.5%			

## Data Availability

Not applicable.
